# Baron Larry’s Medical Campaigns

**Published:** 1845-04

**Authors:** 


*Baron Larrey's Medical Campaigns.*—The Medico-Chirurgical Review, for January, contains a notice of the last published volume of Baron Larrey's Surgical Memoirs, from which we take the following memoranda. They relate to the most distinguished of the patients of that veteran surgeon, and are prefaced by these words:

"The history of their wounds may, I think, occupy a few pages that will prove not without interest or value for the study of those memorable wars which France had to sustain against all the powers of Europe, from 1792 to 1815."

"General Almeras was severely wounded at the battle of the Pyramids. A musket ball traversed the pelvis from before backwards, passing between the testicles, perforating the neck of the urinary bladder, and also the rectum, and escaping from the inner part of the right hip. In spite of the dangerous nature of the wound, this brave officer recovered, and eventually served in the campaigns of Germany, Russia, and France. This case is certainly one of the most remarkable that I ever met with in practice.

"General Andreossy suffered severely from the endemic ophthalmia in Egypt; but recovered perfectly. He died in his native town of Castelnaudari.

"General Arrighi, Duke of Padua, was wounded in the neck by a musket ball at St. Jean d'Acre. The right carotid artery was wounded; and he must inevitably have died of hæmorrhage if a soldier had not had the presence of mind to introduce his two fore-fingers into the wounds, until I arrived and tied the vessel. During the operation, a shell exploded over our heads; but, by a sort of miracle, none of the fragments struck us. The recovery was complete, and the general is still alive.

"Prince Eugene Beauharnais was wounded by a ball in the right temple, at the seventh assault of the same fortress, St. Jean d'Acre. General Bertrand, too, was similarly wounded at the first battle of Aboukir. It was on this occasion that Napoleon first became acquainted with this most faithful and devoted of his servants.

"Marshal Bessieres, Duke of Istria, received a severe contusion of the thigh in the celebrated battle of Wagram; his horse was shot under him at the time. This great warrior was subsequently killed by a cannon ball, while reconnoitring the enemy's lines on the evening before the battle of Lutzen.

"General Blaniac received, at Aboukir, a gun-shot wound in the right side, of the chest. The ball had passed from before backwards, following the direction of the third sternal rib to the seventh, which was fractured in its posterior third; the middle lobe of the lung was injured, and the intercostal artery lacerated. There was a good deal of hæmorrhage from the anterior wound. Symptoms of sanguineous effusion into the chest subsequently occurred, and the operation of *paracentesis thoracis* was performed by enlarging the wound behind. Upwards of a *litre* of dark-colored fluid was discharged. By the most assiduous care, continued for three or four months, this meritorious officer quite recovered his health.

"The only real wound that General Buonaparte (so he is styled here by our author) ever received in his long career of danger, proceeded from a kick of the first Arab horse which he mounted, on leaving the Desert of Lybia. The contusion was followed by an extravasation of blood, to which I gave vent by a small incision. The wound quickly healed. (We have surely read somewhere that Napoleon received, in one of his Austrian campaigns, a contused wound of the leg, that proved, however, to be of no great severity, although great alarm was at first occasioned in the army by the rumor of it.*

General Caffarelli had his right elbow shattered at the siege of St. Jean d'Acre; he was overturned by the blow and fell heavily on his right side. The arm was immediately amputated, and everything went on favorably till the twenty-first day, when symptoms of hepatitis made their appearance. A few days afterwards he died. On dissection, several large abscesses were found in the liver; one of these had burst into

* At Ratisbon, according to Sir W. Scott, he was struck on the foot by a spent musket ball, which produced only a slight contusion.—*Ed.*

the abdominal cavity. The suppurative inflammation had doubtless been induced by the fall.

"General Champeau was severely wounded by a cannon ball at Waterloo. Before I could reach him, a sudden charge of the English cavalry obliged me to withdraw to a considerable distance; and the poor wounded man, like many others, died on that disastrous field without relief.

"General Cheminau had his right leg disorganized by a cannon-shot, at the battle of Lutzen. Although the injury of the soft parts and bones extended close up to the knee, yet, as the joint itself appeared to be intact, I determined to amputate the limb immediately below it. Having made a flap, I disarticulated the head of the fibula, and sawed the tibia across, directly below the attachment of the capsular ligament. I was then surprised to find that the two condyles of the bones were separated by a vertical fracture; the ligament, however, remained uninjured, and there was no symptom of extravasation within the joint. A uniform and circular compression was maintained around the condyles of the tibia, and the dressing of the stump was finished by the application of my unremovable bandaging. This was not removed for nine days, and then immediately reapplied. The success was complete.

"General Coutel, commanding the company of the æronauts attached to the army of Egypt, and who, by means of ascending in a balloon, had ascertained the position of the enemy's army before the celebrated battle of Fleurus, received a musket ball in his right arm at St. Jean d'Acre. Although the humerus was broken, and the injury of the soft parts severe, I determined to try to save the limb. The ends of the bone did not, however, unite, and a false articulation was formed.

"General Count Daboville, now a peer of France, was wounded most severely in the right shoulder at the terrible battle of Wagram. This was one of the fourteen amputations at the shoulder which I performed on that day! Of these cases, twelve recovered perfectly; one patient was killed accidentally by being thrown out of the *ambulance;* and another died of hæmorrhage during the subsequent evacuation of Vienna.

"General Damas was killed by a cannon-shot which struck his chest, at the battle of Moskowa. He was one of the forty general officers who either fell or were most severely wounded on that dreadful day.

"General Count Danthourd was wounded at the battle of

Pesth (1805) by a musket ball, which, after breaking his spur, doubtless penetrated into his right tarsus, and there lodged deeply. Imagining that his wound was only a flesh one, he applied a piece of wetted rag to his foot, and straightway continued his march to the field of Austerlitz, at the glorious battle of which he was present. His wound healed; and he experienced no inconvenience in the foot until fifteen years afterwards, when he began to feel sharp pains in a tumor as big as an olive, which was situated on the dorsal region of the tarsus. Two of the leading surgeons in Paris considered it to be an exostosis; and the general himself was not aware that any ball had ever lodged in the part. On his showing it to me I soon recognised what it was, and told him how he might get rid of it. The ball on extraction proved to be angular, and was lodged in a fibrous cyst.

"General Desaix was wounded, in one of the combats before the capture of the lines at Weissembourg, by a ball which perforated his left cheek. He was killed at the battle of Marengo, 'dont il fixa la victoire.'

"The Duke Deselignac received a gun-shot wound in the left foot and ankle-joint in the last combat of July, 1830. The limb should unquestionably have been amputated at the time; but this was not done, and, on the seventeenth day after the accident, symptoms of tetanus supervened. On being called into consultation, I recommended that the limb should be amputated; but this proposal was not acceded to by the other surgeons in attendance, who, in consequence of the inflamed state of the leg, dreaded gangrene of the stump coming on. The operation was, however, performed early the next morning. The edges of the wound were simply approximated, and kept so by means of lint smeared with a layer of styrax ointment; this position was facilitated by my having made, during the operation, two perpendicular incisions, one over the crest of the tibia, and the other on the opposite side. *The first dressing was not removed for nine days*, and then the wound was found to be suppurating freely; it was entirely healed by the thirty-first day after the operation.

"General Dulong received a gun-shot wound in the right axilla in the Polish campaign of 1807. The ball had passed through the tendon of the pectoral muscle, and the plexus of nerves, grazing the shoulder joint. Although there was scarcely any hæmorrhage, there was good reason to believe that the axillary artery had been divided. Some years subsequently this officer called upon me for my advice. I then learned that, immediately upon the receipt of the wound, the extremity had been stricken with a paralytic torpor and a sense of most distressing coldness, and that no pulse could be felt at the wrist. It had been unwisely determined to try to save the limb. The consequence was, that the hand and arm remained quite paralyzed, and became so atrophied as to resemble a part of a mummy rather than of a living man. I recommended him to submit to amputation; but he would not. His infirmities increasing, he committed suicide.

"Marshal Duroc, Duke of Friuli, was wounded in the right groin, by a piece of a shell, at St. Jean d'Acre. At the close of the battle of Wurtschen, 1813, a cannon ball, which had cut the body of General Kirschner (who was riding at his side) in twain, grazed him in the belly. A large portion of the abdominal parietes was carried away, and the bowels wounded in several places. He survived about thirty hours in dreadful agony.

"General Foy was under my care in his last illness, the disease being cancer of the stomach. 'Had his medical attendants made use of the moxa, as I recommended, his life might probably have been saved, as I have succeeded in doing so in not a few cases in private practice.'

"General Baron Ganan was wounded at the battle of Dresden in the occiput. It was necessary to apply the trephine to remove some portions of the depressed bone; ultimately he recovered. 'A phenomenon, little known to medical men, was often noticed by us in this case. When the ear was applied to the cicatrix over the perforation, the *bruissement* of the cerebral arteries was distinctly perceptible; and the general himself could hear the sounds of the voice when directed to the seat of the wound—a circumstance that still continues, and therefore clearly shows that the opening in the cranium is not yet entirely closed.' (Is there anything wonderful in this?)

"Marshal Grouchy received several severe contused wounds in the thighs and legs at the battles of Moskowa and Craoune. In the case of his wife, Madame la Marechale, I excised the left mamma for a cancerous tumor. This lady quite recovered. she subsequently died very suddenly from a sort of spasmodic cholera, or acute neurosis.

"General Kleber received, at the capture of Alexandria, a ball that struck him in the right temple, divided the integuments, and grazed the parietal bone. I dressed the wound at the base of Pompey's Pillar, and he was sent on to Alexandria. He was subsequently killed at the battle of Heliopolis, where I was exposed to the greatest danger in the discharge of my professional duties.

"Marshal Lannes, Duke of Montebello, was struck by a musket ball, in the right leg, at the battle of Aboukir. Symptoms of tetanus threatened to come on, but were fortunately subdued. He returned with his colleague, Murat, to France, a few weeks after General Buonaparte. He had been wounded also in the temple at the thirteenth assault of St. Jean d'Acre. Some years afterwards he received several wounds in various engagements in Spain. At the famous battle of Essling (1809) his right leg was shattered by a cannon ball, which passed through the knee-joint and wounded the flesh of the left thigh. Immediate amputation was performed by me; and he was sent on to Ebersdorf, where he caught the typhus fever, which prevailed at that time in the army; he died on the thirteenth day after the battle.

"General Lawless—once a professor of physiology in Dublin, who commanded the third foreign regiment, consisting almost entirely of Irishmen—had his left leg shot away by a cannon ball at Lovemberg, on the frontiers of Bohemia. I amputated the limb immediately below the head of the tibia; and, as the army were retreating back upon Dresden at this time, I advised my patient to ride direct to his own home in France, without doing anything to the dressings of his wound, except sponging their surface daily, and keeping the stump enveloped in a piece of linen cloth, or a sheep's skin. By following my directions, he rode on horseback the entire distance from the scene of action to his residence in Tours, having his stump all the while suspended by a belt that passed over his shoulders, and without having it dressed once. On removing the apparatus, when he reached his journey's end, the wound was nearly healed. As a mark of his gratitude, the general made me a present of a magnificent English engraving of Hunter.

"Baron Menou, the third in command of the Egyptian army, was seized with a sporadic plague, just before our debarkation to France; already there were three carbuncles on the right leg, and an incipient one had appeared in the right groin. Unwilling to make the captain of the English frigate (Dido)—which was to convey us home—acquainted with the real facts, I had the general strictly confined to his own cabin, and I then excised the carbuncles,* administering internally camphor,

* This appears to us very extraordinary practice; and yet the baron continued in after life to be so convinced of its propriety—as we have seen nitre, bark, and laudanum. The wounds suppurated freely, before they healed. He was completely cured when we reached Toulon.

"General Viscount Mermet, in consequence of a fall upon his loins in one of the last actions in Italy under Prince Eugene, became entirely paraplegic; the palsy being accompanied with incontinence of the urine. Cupping in the first instance, and subsequently the repeated (twenty times) application of moxas restored him to perfect health, so that he was able to resume active service.

"General Mireur, after having been wounded in the right shoulder at the glorious battle of the Pyramids, was murdered a few days afterwards by the Arabs, while conveying the orders from Buonaparte to Admiral Brueys, to weigh anchor and proceed direct to Corfu, for the purpose of taking in fresh troops and bringing them to Egypt. The death of this young officer was the cause of the destruction of our fleet at Aboukir, and of our subsequent loss not only of Malta, but also of Egypt, which would otherwise have become one of the richest and most beautiful of French colonies.

"Marshal Moncey, Duke of Cornegliano, became affected, in 1830, with an enormous hydrocele. I performed an operation and effected a radical cure.

"General Netherwoot, in one of the combats in Syria, received, from the hands of one of the Mamelukes, a sabre-cut in the right thigh; the entire thickness of the extensor muscles was divided down to the bone, and even this was deeply notched. The wound was very large as well as deep. While one of my assistants kept the limb completely extended, I passed ten stitches from the bottom of the wound through the flesh and integuments, so that the edges might be retained in exact apposition throughout the entire depth. After being bandaged, the limb was placed in a fracture-box, and maintained in a state of extension. This excellent officer was subsequently killed in the expedition to St. Domingo.

"General Count Pajol was wounded in the left fore-arm at the battle of Moskowa. As both bones were broken, and the soft parts much injured, many surgeons would unquestionably have recommended immediate amputation; but I determined to try to save the limb. After having freely *debridé* the wound, and removed all the fragments of bones, I brought the edges together, and applied my apparatus, which I rendered immoin a preceding page—he actually recommended its adoption to the physicians in Dublin.

vable, the fore-arm being kept bent and suspended in a sling. This distinguished warrior accompanied the march of the troops during the dreadful retreat from Russia; and, althongh the wound was not dressed above five or six times, it eventually cicatrized completely, without the movements of the limb being at all impeded.

"General Silly was wounded by a cannon ball in the right knee, at the second battle of Aboukir. I had scarcely completed the amputation of the limb, when a charge of the English cavalry came down upon us. I took the wounded man upon my shoulders, and carried him along a very uneven road to the rear-guard of our army. I arrived at Alexandria with my honorable burden, and had the satisfaction there of completing the cure.

"Marshal Soult was never dressed by me on the field of battle; but I attended him in Paris for a severe contusion of the leg, which had been fractured. A traumatic sanguineous tumor supervened; this required a delicate operation should be performed. The success was complete, and my illustrious patent speedily recovered.

"Marshal Suchet, Duke of Albufera, on returning from his most arduous campaigns in Spain, consulted me for a cancerous affection of the stomach, the precursory symptoms of which had already appeared. The disease, although it had been very sensibly arrested, finally proved fatal.

"Marshal Victor, Duke of Belluno, was wounded in the right thigh at the battle of Craoune, (1814.) The ball had passed between the femur and the femoral artery, which I found to be partly denuded; the sciatic nerve was injured. The two wounds were immediately 'debridés,' and subsequently dressed after my usual plan. The marshal continued to suffer from a traumatic neuralgia to the end of his life, in spite of every remedy that was tried.

"General Zayonchek, seventy-five years old, was wounded in the knee at the passage of the Beresina. The wound required the amputation of the limb. I performed the operation under the cannon of the enemy, and while there was a very heavy fall of snow. To prevent my being incommoded by 'ce meteore,' two general officers held the patient's cloak over our heads. He returned to Warsaw, his native place, and finally recovered. He was subsequently made a prince, and the viceroy of Poland, by Emperor Alexander. He died at the advanced age of eighty-six years."

